# Commentary: An evaluation of traditional Persian medicine for the management of SARS-CoV-2

**DOI:** 10.3389/fphar.2023.1124157

**Published:** 2023-02-02

**Authors:** Haniye Mirhosseini, Jale Aliasl, Fatemeh Eghbalian

**Affiliations:** ^1^ Department of Traditional Medicine, Institute for Studies in Medical History, Persian and Complementary Medicine, Iran University of Medical Sciences, Tehran, Iran; ^2^ Department of Traditional Medicine, School of Persian Medicine, Iran University of Medical Sciences, Tehran, Iran; ^3^ Student Research Committee Iran University of Medical Sciences, Tehran, Iran; ^4^ Traditional Medicine Clinical Trial Research Center, Shahed University, Tehran, Iran

**Keywords:** SARS-CoV-2, traditional Persian medicine, cardio tonic, infection, herbal medicine

We read with interest the article by Bahramsoltani and Rahimi published in November 2020 (Bahramsoltani and Rahimi, 2020). They reviewed the pharmacological mechanisms of medicinal plants that could affect SARS-CoV-2 infection and related organ damage according to Traditional Persian Medicine (TPM). Bahramsoltani and Rahimi first introduced several medicinal plants with potential benefits for treating SARS-CoV-2 infection according to TPM textbooks. Then, they reviewed pharmacological studies in the literature that examined medicinal plants, and discussed their mechanism of action in SARS-CoV-2 infection (Bahramsoltani and Rahimi, 2020). Most of the medicinal plants included in this review show multitargeted activity and protective mechanisms in the tissues damaged in SARS-CoV-2 infection. The most important effects of the medicinal plants and their isolated phytochemicals include anti-inflammatory activity, immunomodulatory effect and antioxidant properties. This review is very update and in a meticulous way was written. While thanking the authors for presenting a scientific and valuable article, we would like to mention a few points about this article, and complement their findings. Table 1 in their article failed to present some of the medicinal plants in the TPM reference books with protective properties against humor infection and excitation. Some of these plants are as follows.

1. Zedoary (*Curcuma zedoaria*) has cardiotonic and nephrotonic properties and prevents humor infection (Aghili Khorasani, 1771).
2. Lemon (*Citrus limon*) is mentioned in Makhzan-al-Adviah for preventing excitation of blood and bile (Aghili Khorasani, 1771).
3. Chamomile (*Matricaria chamomilla*) is mentioned in Makhzan-al-Adviah for preventing black bile and phlegm (Sauda & Balgham) infection (Aghili Khorasani, 1771).
4. Cinnamon (cinamum zelinicum) is cardiotonic, and prevents humor infection as stated in Makhzan-al-Adviah and Canon of Medicine (Qanoon-fil-Tib) (Aghili Khorasani, 1771; Avicenna, 1025).
5. Green olive oil (Olea europaea) (Zit al-Anfaq) disrupts humor infection as mentioned in TPM references (Aghili Khorasani, 1771).
6. Zaferān (*Crocus sativus* L.), mentioned in the Makhzan-al-Adviah, cures phlegm infection, as well as preventing and protecting it from change and corruption (Aghili Khorasani, 1771).
7. Nepeta mentoides or lavender is cardiotonic, and prevents humor infection, as stated in Makhzan-al-Adviah and Canon of Medicine (Qanoon-fil-Tib) (Aghili Khorasani, 1771; Avicenna, 1025).

In addition, wewould like to add another information to the original manuscript, and complement their findings.

Ghār, Barg-e-Bou (*Laurus nobilis* L.) was also mentioned in Table 1 as an agent that prevents humor infection (Bahramsoltani and Rahimi, 2020); however, we did not find this property in the review of the mentioned reference book (Aghili Khorasani, 1771).

In conclusion, we thank the authors of the article “An Evaluation of Traditional Persian Medicine for the Management of SARS-CoV-2,” and heartily recommend that they appropriately modify it to strengthen their findings.
