# Corrigendum: Commentary: An evaluation of traditional Persian medicine for the management of SARS-CoV-2

**DOI:** 10.3389/fphar.2023.1296550

**Published:** 2023-09-28

**Authors:** Haniye Mirhosseini, Jale Aliasl, Fatemeh Eghbalian

**Affiliations:** ^1^ Department of Traditional Medicine, Institute for Studies in Medical History, Persian and Complementary Medicine, Iran University of Medical Sciences, Tehran, Iran; ^2^ Department of Traditional Medicine, School of Persian Medicine, Iran University of Medical Sciences, Tehran, Iran; ^3^ Student Research Committee Iran University of Medical Sciences, Tehran, Iran; ^4^ Traditional Medicine Clinical Trial Research Center, Shahed University, Tehran, Iran

**Keywords:** SARS-CoV-2, traditional Persian medicine, cardio tonic, infection, herbal medicine

In the published article, there was an error in the author list, and author **Fatemeh Eghbalian** was erroneously excluded. The corrected author list appears below.

“Haniye Mirhosseini^1,2,3^, Jale Aliasl^4^ and Fatemeh Eghbalian^1,2,^*”

The Author Contributions were updated to include the contributions of the author, Fatemeh Eghbalian. The correct Author Contributions appear below.

“HM and FE searched the literature. HM, FE, and JA conceived and wrote the manuscript. All authors contributed to drafting the manuscript and approved the submitted version.”

The authors apologize for these errors and state that this does not change the scientific conclusions of the article in any way. The original article has been updated.

